# Perineuronal nets in neurodegeneration

**DOI:** 10.18632/oncotarget.13420

**Published:** 2016-11-16

**Authors:** Sighild Lemarchant, Sara Wojciechowski, Jari Koistinaho

**Affiliations:** A. I. Virtanen Institute for Molecular Sciences, Biocenter Kuopio, University of Eastern Finland, Kuopio, Finland

**Keywords:** perineuronal net, chondroitin sulfate proteoglycans, amyotrophic lateral sclerosis, Alzheimer’s disease, neurodegeneration

At the end of the 19^th^ century, Camillo Golgi discovered that neurons were enwrapped by a net, but it was only recently that neuroscientists started to intensely investigate the role of this mysterious reticular structure. Today, perineuronal nets (PNNs) are known to be lattice-like extracellular matrix aggregates surrounding synapses at the soma, proximal axons and dendrites of several types of neurons, including fast-spiking GABAergic interneurons, pyramidal neurons, cerebellar Purkinje cells and spinal cord motoneurons. PNNs are composed of chondroitin sulfate proteoglycans (CSGPs, including aggrecan, neurocan and brevican), hyaluronan, tenascins and link proteins. They first appear at the end of the critical period of neurodevelopment when they are thought to contribute to the constitution and stabilization of synapses around mature postsynaptic neurons. In adults, PNNs are stable and restrict neuroplasticity, but have an ability to undergo dynamic changes in response to learning and memory, stress, activation of the immune system, or CNS injuries and diseases. This remodeling is achieved by CSPG-degrading proteases, such as matrix metalloproteinases and ADAMTS proteoglycanases (a disintegrin and metalloproteinase with thrombospondin motifs) [1].

Alterations in the PNN structure have been observed in several CNS diseases, including psychiatric disorders, Alzheimer’s disease (AD) and amyotrophic lateral sclerosis (ALS). Recent studies have shown that a loss of PNNs is associated with neurodegeneration and progression of ALS-related symptoms in rodents over-expressing the human superoxide dismutase SOD1^G93A^ mutation. Forostyak and colleagues reported that the PNNs surrounding cervical and lumbar spinal cord motoneurons of SOD1^G93A^ rats are considerably degraded by the end-stage of the disease when compared to wild-type rats. Interestingly, they demonstrated that transplantation of mesenchymal stromal cells from the bone marrow partially prevents ALS-related degradation of PNNs in SOD1^G93A^ rats, thereby increasing motoneuron survival and lifespan [2]. Similarly, we recently showed a decrease in PNNs around lumbar spinal cord motoneurons of SOD1^G93A^ mice at the symptomatic stage. The expression and proteolytic activity of ADAMTS-4, the highest expressed ADAMTS proteoglycanase in the CNS, were progressively decreased in the spinal cord during advancement of ALS pathology. Also, when active recombinant human ADAMTS-4 was intracerebroventricularly injected into SOD1^G93A^ mice before the disease onset, the ALS-induced degradation of PNNs was associated with enhanced neurodegeneration and neuromuscular dysfunctions. The study indicated that decreased endogenous activity of ADAMTS-4 may be an adaptive attempt to preserve integrity of PNNs in ALS [3]. Altogether, these studies suggest that the disorganization/ degradation of PNNs observed during the progression of ALS may contribute to motoneuron degeneration and disease severity [2, 3]. Several studies by Morawski and collaborators have described a massive loss of PNNs in cortical and subcortical brain areas of patients with AD and in AD mouse models [1]. How exactly do PNNs protect neurons in AD or ALS remains an open question, but compelling evidence suggests a function of PNNs as being a chemical and physical barrier against the hostile environment common for neurodegenerative diseases. Indeed, transgenic mice lacking PNNs components, such as aggrecan, link protein 1, and tenascin R, are far more prone to the neuronal death induced by iron injection-induced oxidative stress than their wild-type counterparts [4]. In the context of AD, neurons ensheathed by a net were protected from neurofibrillary tangles, phosphorylated Tau and lipofuscin accumulations contrary to neurons without PNNs [1]. Additionally, cortical neurons deprived of PNNs after treatment with chondroitinase ABC, an enzyme capable of removing chondroitin sulfate chains from CSPGs, were susceptible to amyloid-β^1-42^ toxicity *in vitro,* whereas cortical neurons with intact PNNs were not [5]. Overall, CSPGs seem to play a central role in the neuroprotective effect of PNNs by binding iron and toxic molecules, thereby preventing them from entering neurons and causing irreversible damage.

The role of PNNs was ignored for a very long time since their discovery by Camillo Golgi. Currently, PNNs are now considered a crucial element in the “tetrapartite” synapse. PNNs may defend neurons against oxidative stress, lipofuscin accumulation and amyloid-β toxicity. Therefore, degenerative alterations in PNNs may compromise synapse integrity and stability, which may eventually contribute to progression of neurodegenerative diseases, such as AD and ALS. The involvement of PNNs degradation in neurodegeneration should be carefully taken into account in emerging studies aiming at promoting long-term memory in AD by using chondroitinase ABC [6]. Therapeutic treatments directed to preserve or reconstruct the integrity of PNNs may represent a key strategy to protect neurons against neurodegenerative processes.
